# The regulatory role of microRNAs in common eye diseases: A brief review

**DOI:** 10.3389/fgene.2023.1152110

**Published:** 2023-03-29

**Authors:** Javier A. Benavides-Aguilar, Jonathan I. Morales-Rodríguez, Héctor Ambriz-González, Luis M. Ruiz-Manriquez, Antara Banerjee, Surajit Pathak, Asim K. Duttaroy, Sujay Paul

**Affiliations:** ^1^ Tecnológico de Monterrey, School of Engineering and Sciences, Queretaro, Mexico; ^2^ Tecnologico de Monterrey, School of Medicine and Health Science, Monterrey, Mexico; ^3^ Chettinad Academy of Research and Education (CARE), Chettinad Hospital and Research Institute (CHRI), Department of Medical Biotechnology, Faculty of Allied Health Sciences, Chennai, India; ^4^ Department of Nutrition, Institute of Basic Medical Sciences, Faculty of Medicine, University of Oslo, Oslo, Norway

**Keywords:** microRNA, cataracts, glaucoma, macular degeneration, uveitis

## Abstract

MicroRNAs (miRNAs) are highly conserved, small non-coding RNA molecules (∼21 nucleotides) that regulate numerous biological processes, including developmental timing, hematopoiesis, organogenesis, apoptosis, cell differentiation, and proliferation either by mRNA degradation or translation repression. Since eye physiology requires a perfect orchestration of complex regulatory networks, an altered expression of key regulatory molecules such as miRNAs potentially leads to numerous eye disorders. In recent years, comprehensive progress has been made in demonstrating the precise roles of miRNAs, emphasizing their potential use in diagnostic and therapeutic purposes of chronic human diseases. Thus, this review explicitly illustrates the regulatory roles of miRNAs in four common eye disorders, such as cataract, glaucoma, macular degeneration, and uveitis, and their application in disease management.

## 1 Introduction

The eyes are the primary organs for vision and are composed of different structures. The eyeballs in orbit comprise up to 20% of the orbital volume, whereas the remaining 80% is occupied by extraocular muscles, fat, nerves, blood vessels, fascia, and the lacrimal gland (Galloway et al., 2016). Degenerative modifications in the structure of the eyes are currently a relevant cause of blindness (Galloway et al., 2016). Among eye diseases, cataracts, glaucoma, and corneal opacities are the most common (World Health Organization, 2022). A cataract is the leading cause of reversible blindness, whose prevalence is higher in lower socioeconomic status since the most effective treatment option, surgical removal of the lens usually unaffordable to most of the population (Lam et al., 2015). Similarly, glaucoma, a chronic optic nerve disorder, often leads to blindness if it remains untreated; while the most conventional treatment, laser therapy, might lead to complications of the pupil (Schuster et al., 2020). Corneal opacities, such as macular degeneration and uveitis, are a group of disorders that impede the cornea’s normal functioning of light transmission; treatments such as corneal transplantation can be performed; nevertheless, this can involve risks and delayed rehabilitation (Dohlman et al., 2019; Sharma et al., 2019). Currently, over 65 million people worldwide are suffering from cataracts, whereas nearly 80, 196, and 2.3 million people have glaucoma, macular degeneration, and uveitis, respectively (Bright Focus Organization, 2021; The Ocular Immunology and Uveitis Foundation, 2022; World Health Organization, 2022).

MicroRNAs (miRNAs) are small (∼21 nucleotides in length) non-coding RNA molecules involved in the post-transcriptional regulation of cellular signaling pathways. Since the discovery of miRNAs in *C. elegans* by Lee et al. (1993), they have been identified in several organisms, including humans. This tiny molecule has the ability to regulate several biological processes, including cell growth, differentiation, development, and death (Raghunath and Perumal, 2015). For example, several studies have demonstrated that miRNAs are involved in cell cycle progression by directly targeting different cell cycle-associated transcript effectors (e.g., cyclins, CDKs, and CDKIs) or indirectly by modulating different genes responsible for cell cycle control (Mens and Ghanbari, 2018; Rodrigues et al., 2018; Ghafouri-Fard et al., 2020). Moreover, miRNAs can also profoundly impact the expression of various cell death-related genes such as pro- and antiapoptotic genes, autophagy regulation genes, endoplasmic reticulum (ER) stress genes, or necroptosis-related genes evidencing a complex regulatory network between miRNAs and cell cycle/cell death pathways (Shirjang et al., 2019; Jang and Lee, 2021). Furthermore, it has been demonstrated that human miRNAs regulate approximately 60% of protein-coding genes associated with several cellular processes. Their altered expression can significantly lead to the development of a wide range of human pathologies, thus making them a promising disease biomarker and novel therapeutic targets (Hanna et al., 2019; Ruiz-Manriquez et al., 2022a; Ruiz-Manriquez et al., 2022b; Bravo-Vázquez et al., 2023).

MiRNA biogenesis in humans begins in the nucleus when their corresponding genes are transcribed into a hairpin structure known as primary transcript/miRNA (pri-miRNA). Subsequently, the pri-miRNA is processed by Drosha and DGCR8 into precursor miRNA (pre-miRNA) and transported to the cytoplasm by Exportin 5. Afterward, RNA Dicer and the RNA-binding protein TRBP further process the pre-miRNA into a short miRNA/miRNA* duplex, from which mature miRNA forms the RNA-induced silencing complex (RISC) with the aid of Argonaute 2 protein (AGO2) and recognizes a specific mRNA by a sequence complementary triggering translational inhibition or mRNA degradation (Paul et al., 2021; Ruiz-Manriquez et al., 2022a; Ledesma-Pacheco et al., 2023). The majority of research suggests that miRNAs cause the inhibition of translation, deadenylation of mRNA, and decapping by binding to a particular sequence located in the 3′UTR of their target mRNAs (Dexheimer and Cochella, 2020). Despite this, miRNA binding locations have also been discovered in other areas of the mRNA, such as the 5′UTR, coding sequence, promoter regions, and other mRNA regions (O’Brien et al., 2018). Although miRNA binding to the promoter region has been reported to induce transcription, it has been shown that miRNA attachment to the 5′UTR and coding regions can silence the expression of specific genes in a context-dependent manner (Treiber et al., 2019).

It has also been stated that a single transcript might be targeted by multiple miRNAs, while a sole miRNA can regulate up to 200 different mRNAs (Ruiz-Manriquez et al., 2022c; Iacomino, 2023). The investigation of how miRNA-mediated regulation is influenced by factors such as miRNA abundance/localization, cell type, and cell state is currently being extensively studied (Vishnoi and Rani, 2023); however, it has been hypothesized that an aberrant expression of these molecules influences critical biological processes leading to various pathological outcomes associated with diseases (Diener et al., 2022).

Studies showed that several miRNAs present in eye tissues have crucial roles in normal eye functioning through involvement in processes such as cell growth and proliferation, differentiation, and apoptosis (Raghunath and Perumal, 2015; Vishnoi and Rani, 2017), and their dysregulation hinders the normal functions of the associated target proteins leading to the pathogenesis of several (Table 1) eye diseases (Liu et al., 2019a; Li et al., 2019b; Liu et al., 2020a; Elshelmani et al., 2021; Liao et al., 2022). Hence, in this current review, we addressed the role of different miRNAs in the 4 most common eye diseases, such as cataract, glaucoma, macular degeneration, and uveitis, to explore their potential in the prognosis and treatment of those diseases.

**TABLE 1:** miRNAs involved in different common eye diseases.

Disease	miRNA and expression pattern	Target gene	Biological mechanism	Sample	References
**Cataract**	↓ miR-199a-5p	SP1	Activation of EMT in diabetic LECs	Human hyperglycemic LECs	Liu et al. (2020b)
	↑ let-7g-5p	No reports	No reports	Human LECs	Kim et al. (2021)
	↑ miR-23a-3p		Induction of ROS formation		
	↑ miR-23b-3p		Induction of ROS formation and induction of autophagy		
	↑ miR-125a-5p		No reports		
	↓ let-7a-5p				
	↓ let-7d-5p				
	↓ miR-22–3p		Induction of autophagy		
	↓ miR-16–5p		Induction of apoptosis and autophagy		
	↑ miR-551b	CRYAA	Reduction of HLEC viability	HLECs	Gao et al. (2021a)
	↓ miR-29b	CACNA1C	Induction of apoptosis	Exosomes isolated from AH	Gao et al. (2021b)
	↑ miR-15a	BCL2/E2F3	Suppression of cell growth and an increase in apoptosis	HLE-B3 cells	Li et al. (2019a)
**Glaucoma**	↑ miR-210–3p	No reports	No reports	Serum	Liu et al. (2019b)
	↑ miR-93	NFE2L2	Induction of apoptosis	GTM cells	Wang et al. (2016)
	↑ let-7a-5p	Crosstalk with BCL-xL	Induction of apoptosis due to toxic autophagy	AH	Seong et al. (2021)
	↑ let-7c-5p	No reports	Neurogenesis, aging, apoptosis, and autophagy		
	↑ let-7f-5p				
	↑ miR-192–5p				
	↑ miR-10a-5p				
	↑ miR-10b-5p				
	↑ miR-375	BDNF			
	↑ miR-143–3p	No reports			
**Macular degeneration**	↑ miR-16–5p*	No reports	No reports	Plasma	Ulańczyk et al. (2019)
	↑ miR-17–3p	PIGF			
		PIGF acidic			
	↑ miR-17–5p	FGF acidic			
		Endostatin			
	↑ miR-23a-3p	Endostatin			
	↑ miR-126–5p	No reports			
	↑ miR-93				
	miR-150^§^				
	miR-146a^§^	Endostatin			
	↑ miR-223–3p				
	↓ miR-21–3p	No reports			
	↓ miR-155–5p				
	miR-23a-3p*^§^				
	miR-30b*^§^				
	miR-191–5p*^§^				
	↑ miR-486–5p	IGF1/AKT/mTOR pathway CD40 pathway, mTOR signaling pathway	Angiogenesis, inflammatory response, and photoreceptor degeneration	Blood serum	Elbay et al. (2019)
	↑ miR-626	SLC7A5	Neurodegenerative process		
	↓ miR-885–5p	No reports	No reports		
	↑ miR-19a	NTRK1	Apoptosis, coagulation, neurodegeneration	Blood serum	Elshelmani et al. (2020), Elshelmani et al. (2021)
		IL1R1			
		PRKA2B			
		PIK3CA			
		MAP3K14			
		MAPK1			
		PIK3R3			
		PPP3CANFATC2			
	↑ miR-410	SPHK2			
	↑ miR-126	ITGA6	Neurodegeneration		
		IGF-1			
**Uveitis**	↓ miR-182–5p	TAF15	Inhibition of Th17 cell development	Mouse-derived Th17 cells	Zhang et al. (2020)
		STAT3			
	↑ miR-379–5p	SEMA3A	Decrease of B and T cell activity	Serum of EAU mice	Li et al. (2021)
	↑ miR-146a	CD80	The proliferation of T cells	PBMC	O’Rourke et al. (2019)
		PRKCE	Phosphorylation of proteins		
		VASN	Inhibition of TGFβ		
	↑ miR-155	SMAD2	Promotor of inflammation		
		TYRP1			
		FBXO22			

↑ Upregulated (overexpressed) miRNA.

↓ Downregulated (underexpressed) miRNA.

* Proposed biomarker.

^§^ Differential expression profile not reported.

## 2 Cataract

A cataract is caused by the clouding of the eye lens, which is typically clear (Lam et al., 2015). Despite cataracts being often curable, this is the leading cause of blindness globally. A study in 2020 revealed that out of the 36 million people that were blind worldwide, 13.5 million people developed this condition derived from cataracts (Hashemi et al., 2020). A cataract is an age-related ailment (Age-related cataract/ARC) and increases significantly over the age of 60; nevertheless, it can be a birth condition (Lam et al., 2015). Additionally, external factors such as smoking, regular exposure to UV light, or chronic metabolic diseases, such as diabetes, might influence cataract appearance (Hashemi et al., 2020). It is well established that cataracts can be triggered by diabetes mellitus (DM), leading to diabetic cataracts (DC), which could appear at an earlier age and in a more severe form than ARC (Liu et al., 2020b). The pathogenesis of DC is characterized by the aberrant expression of genes related to the development, autophagy, proliferation, differentiation, and epithelial–mesenchymal transition (EMT) of Lens epithelial cells (LECs) (Liu et al., 2020b). Interestingly, Liu et al. (2020b) noticed that the miR-199a-5p levels were significantly low in DC and in human hyperglycemic LECs. miR-199a-5p targets SP1, a gene whose downregulation is related to the repression of EMT in diabetic LECs *via* diminishing the α-SMA and augmenting the E-cadherin transcription. Studies also found that the unfolding of structural lens proteins crystallins through destabilization, covalent modifications, or age-related damage might lead to the development of cataracts by forming opaque and dense aggregates on the lens (Lam et al., 2015). The only treatment available for cataracts is ultrasonic or manual surgery (Lam et al., 2015).

Significant differential expression of eight miRNAs in LECs was reported by Kim et al. (2021) in various types of ARC (senile cataract), such as cortical cataract, nuclear cataract (NC), and posterior and anterior subcapsular cataract (ASC). They noticed that the expression levels of let-7g-5p, miR-23a-3p, miR-23b-3p, and miR-125a-5p were higher in cataractous samples than healthy controls, while let-7a-5p, let-7d-5p, miR-22-3p, and miR-16-5p were downregulated. They also observed that most of the abovementioned differentially expressed miRNAs are associated with oxidative stress; for example, miR-23 induces ROS formation and promotes cataractogenesis. On the other hand, miR-16 (an autophagy inducer miRNA) deficiency has been linked to apoptosis in tissues due to hyperoxia (Mrowicka et al., 2021). Additionally, ROS production was found to be related to the accumulation of dysfunctional mitochondria in cells that lack autophagy and whose housekeeping role is crucial in differentiating fiber cells of the adult lens. In this context, miR-23b suppresses autophagy activation, while miR-22 and miR-16 promote autophagy (Kim et al., 2021). Understanding the relationship between ROS and autophagy is relevant in cataracts since, in ARC, it has been observed that autophagy is a crucial factor for regulating apoptosis of LECs (Huang et al., 2022).

Differential expression of exosomal miRNAs has also been observed between ARC and DC. For example, in human aqueous humor (AH) samples, miR-551b was upregulated in a DC cohort compared to an ARC cohort. The potential target of miR-551b is CRYAA, whose expression was significantly downregulated in human LECs (HLECs). It was also noticed that mimics of miR-551b reduced the viability of HLECs drastically, and its inhibitors lessened the apoptosis of these cells (Gao et al., 2021a). Additionally, Gao et al. (2021b) found that in exosomes isolated from AH, miR-29b was substantially downregulated in a DC cohort than an ARC cohort, thus triggering the expression of its potential target Calcium Voltage-Gated Channel Subunit Alpha1 C (CACNA1C). High CACNA1C level is associated with increased concentration of Ca^2+^ inducing apoptosis of HLECs in patients with DC (Gao et al., 2021b). The fact that both miR-551b and miR-29b showed dysregulations in DC cohorts compared to ARC cohorts leads to the conclusion that DC and ARC present a differential miRNA profile and, therefore, miRNA expression in cataracts is not homogeneous. Still, it depends on the type of origin of the disease. Likewise, miR-15a is another miRNA associated with the modulation of HLECs apoptosis and proliferation. Li et al. (2019b) reported that HLE-B3 cells treated with H_2_O_2_ showed an upregulation of miR-15a, decreasing BCL2 and E2F3 expression and inducing apoptosis.

**FIGURE 1:**
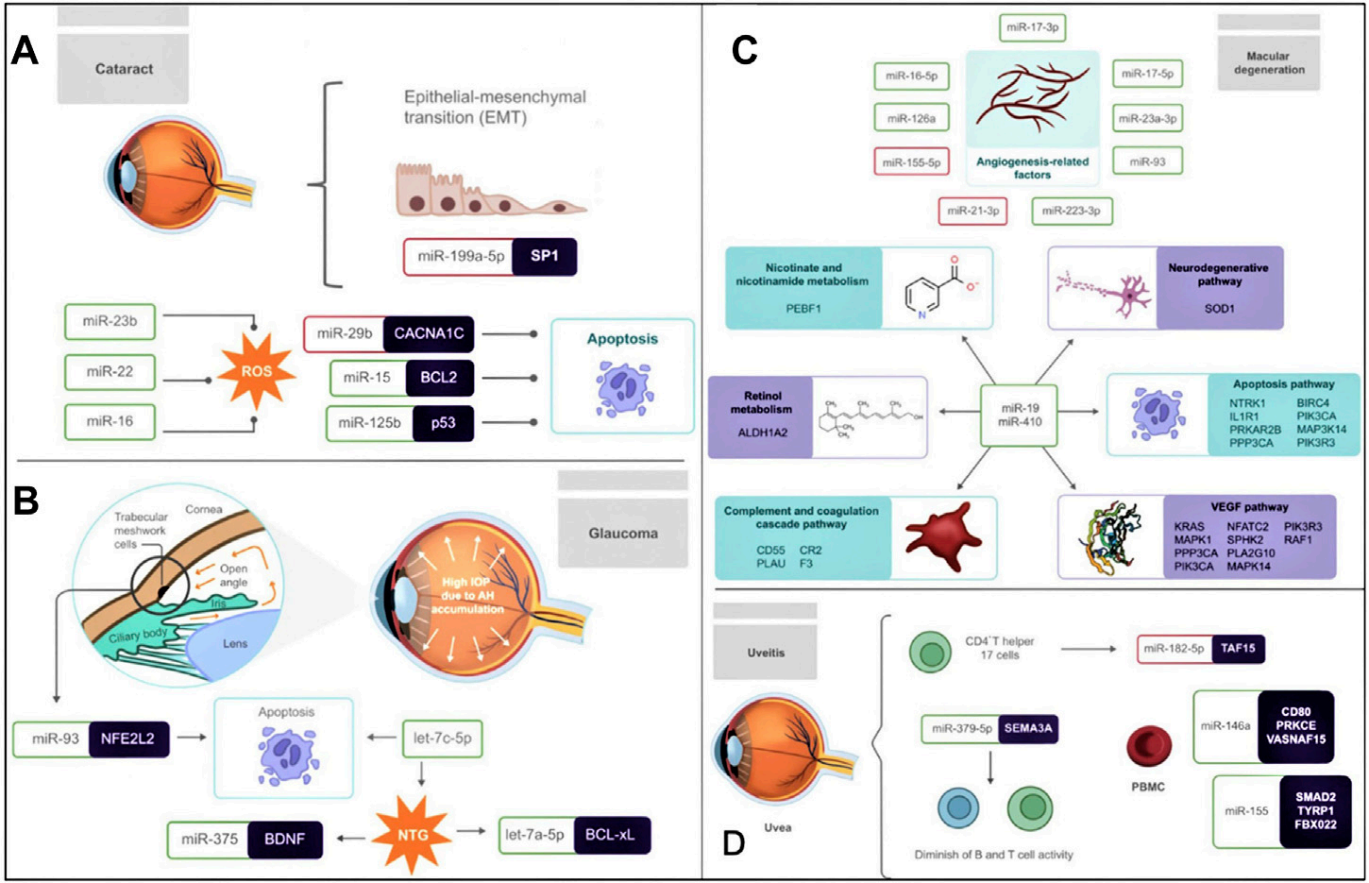
Association of miRNAs in the pathogenesis of **(A)** cataract **(B)** glaucoma **(C)** macular degeneration **(D)** uveitis **(A)** The differential expression of several miRNAs strongly influences Cataractogenesis. miR-199a-5p is downregulated and targets SP1, therefore affecting the EMT of LECs. ROS is another contributing factor to the development of cataracts, and their formation is promoted by the upregulation of miR-23b, miR-22, and miR-16. Also, LECs apoptosis is another crucial biological mechanism influenced by the downregulation of miR-29b and the upregulation of miR-15 and miR-125b, which target CACNA1C, BCL2, and p53, respectively. **(B)** Glaucoma is characterized by the accumulation of AH in front of the eye leading to high IOP. In addition, the upregulation of miR-93, which targets NFE2L2, triggers apoptosis in GTM cells. In NTG, let-7c-5p and let-7a-5p are related to apoptosis, while the targeting of BDNF by miR-375 has been related to neurogenesis, aging, apoptosis, and autophagy. **(C)** In macular degeneration, the levels of angiogenesis-related factors are correlated with the upregulation of miR-17-3p, miR-17-5p, miR-23a-3p, miR-93, miR-223-3p, miR-126a, and miR-16-5p, as well as the downregulation of miR-155-5p and miR-21-3p. MiR-19 and miR-410 are significantly relevant in macular degeneration since they target multiple genes involved in processes such as nicotinate and nicotinamide metabolism, the neurodegenerative pathway, the apoptosis pathway, the VEGF pathway, the complement, and coagulation cascade pathway and retinol metabolism. **(D)** The pathogenesis of uveitis is related to the differential expression of several miRNAs. Underexpressed miR-182-5p in EAU mouse-derived-Th17 cells negatively regulates its development. While the upregulation of miR-146a and miR-155 was observed in PBMC, decreasing the expression of the immunosuppressive cytokine TGFβ, resulting in inflammation. The upregulation of miR-379-5p, which targets SEMA3A, is also linked to decreased B and T cell activity. MiRNAs inside a red box represent downregulation and miRNAs inside a green box represent upregulation.

Differential expression of miRNAs in cells such as LECs implies altering crucial processes, such as cell proliferation, apoptosis, EMT, or ROS formation, which contribute significantly to the cataractogenic process (Figure 1A). Therefore, therapeutic tools, such as miRNA inhibitors, could be a potential alternative treatment for cataracts since processes such as HLEC apoptosis might be repressed.

## 3 Glaucoma

Glaucoma (the second main cause of blindness worldwide) is a severe ocular disorder associated with accumulating AH fluid in front of the eye. It is estimated that the incidence of this disease will increase from 76.5 million in 2020 to 111.8 million by 2040 (Tham et al., 2014). The human trabecular meshwork (HTM) is a cluster of cells and matrix that regulates the AH outflow to control the intraocular pressure (IOP) (Gasiorowski and Russell, 2009), and studies showed that excessive AH increases the IOP leading to irreversible damage of the optic nerve, as well as the loss of retinal ganglion cells and cupping of the optical disc (Weinreb et al., 2014). There are two main types of glaucoma: primary open-angle glaucoma (POAG) and primary angle-closure glaucoma (PACG). POAG occurs when the eye loses the capacity to drain fluids; as a consequence, the excessive IOP aggravates optic nerve degeneration without expressing pain or vision changes (Molasy et al., 2017). On the other hand, PACG is characterized by the absence of space between the iris and the drainage angle, which leads to an acute attack and the expression of several symptoms such as heavy eye pain, blurred vision, dilated pupils, nausea, and IOP above 30 mmHg (Dastjerdi et al., 2021). Several recent reports have demonstrated the association of altered miRNAs with glaucoma and their therapeutic potential.

Aiming to create a circulating miRNAs profile linked to glaucoma, Liu et al. (2019b) analyzed 136 serum samples collected from POAG patients and healthy individuals/control groups, and they noticed that miR-210-3p was consistently and significantly upregulated in POAG samples than the control groups, respectively, suggesting its credibility as a POAG biomarker. Similarly, Wang et al. (2016) intended to evaluate the effect of miR-93 on glaucoma trabecular meshwork (GTM) cells (critical cells that maintain the flow of AH), and they observed that the level of this miRNA was substantially higher in GTM cells as compared to HTM cells. To precisely understand the effect of miR-93 on GTM cells' viability, they modified its expression levels by lentivirus-mediated transfection, and the results revealed that the viability of GTM cells with induced miR-93 expression was considerably decreased post-transfection. In contrast, cells with repressed miR-93 showed promoted cell viability by inhibiting apoptosis. Moreover, they demonstrated that miR-93 triggers apoptosis in GTM cells by targeting NFE2L2, a transcription factor for the expression of cytoprotective genes, leading to glaucoma.

Seong et al. (2021) recently compared the miRNA expression profile from individual AH samples between normal controls and patients with normal-tension glaucoma (NTG). Several relevant miRNAs (let-7a-5p, let-7c-5p, let-7f-5p, miR-192-5p, miR-10a-5p, miR-10b-5p, miR-375, and miR-143-3p) involved in neurogenesis, aging, apoptosis, and autophagy, were found to be substantially upregulated in NTG patients. Among those miRNAs, let-7a-5p, let-7c-5p, and miR-375 are reported to have a concrete biological function in NTG, while the others are the subject of further research (Figure 1B). For example, Let-7a-5p is known to regulate BCL-xL leading to toxic autophagy and ultimately in cell death, while let-7c-5p is associated with the extracellular matrix (ECM)-receptor interaction; additionally, overexpressed miR-375 might regulate negatively the brain-derived neurotrophic factor (BDNF), a member of the neurotrophin family produced by retinal ganglion cells (RGCs) which is crucial for the protection of inner retinal integrity with age (Figure 1B).

## 4 Macular degeneration

Macular degeneration, also known as age-related macular degeneration (AMD), is a common eye condition that affects the macula (the portion of the retina responsible for the central vision acuity), causing severe and permanent visual impairment (Lim et al., 2012). AMD is the leading cause of vision loss in older people (National Eye Institute, 2020) and can be classified into two types, geographic atrophy AMD (dry AMD) and neovascular AMD (wet AMD). Dry AMD is characterized by the formation of large protein deposits in the macula known as “drusen” and accounts for more than 80% of the total cases. On the other hand, wet AMD causes blood, lipids, and fluid leakage due to the abnormal growth of blood vessels in the subretinal region, leading to fibrous scarring. Even though wet AMD accounts for a small percentage of the total AMD cases, vision loss often occurs faster and is responsible for more than 80% of the severe vision loss caused by AMD (Lim et al., 2012; Boyd, 2022). As the name indicates, aging is the major cause of AMD; however, several risk factors such as smoking, obesity, hypertension, and family history might contribute to the development of AMD (Lim et al., 2012; Al-Zamil and Yassin, 2017; World Health Organization, 2019). Even though it is well known that several factors contribute to the development of AMD, recent research has found that miRNAs play crucial roles in the development, progression, and severity of AMD.

Studies revealed that circulating miRNAs could also serve as potential biomarkers for AMD detection (Urbańska et al., 2022; Elshelmani and Rani, 2023), as reported in other chronic diseases, such as cancer (El-Daly et al., 2023), osteoporosis (Messner et al., 2022), epilepsy (Xie et al., 2023), pulmonary hypertension (Düzgün et al., 2023), nephropathies (Sun et al., 2022), and hepatitis (Jiang et al., 2023). Furthermore, the significance of circulating miRNAs in numerous therapeutic areas makes them a promising asset for translational medicine applications (Pozniak et al., 2022). Pathological angiogenesis is an important factor for AMD development, especially in wet AMD, due to the formation of blood vessels that cross Bruch’s membrane and pass to the retinal pigment epithelium (RPE), knocking down photoreceptors leading to vision loss (Vega et al., 2021). In this framework, Ulańczyk et al. (2019) screened 10 angiogenesis-related factors and an array of 14 miRNAs in plasma samples of 354 AMD patients (175 dry AMD and 179 wet AMD) and 121 healthy controls. Results showed that AMD is associated with lower levels of angiogenesis-related factors, such as angiogenin, endostatin, FGF-basic, and PIGF, and higher levels of FGF-acidic, along with upregulated miRNAs such as miR-16-5p, miR-17-3p, miR-17-5p, miR-23a-3p, miR-126-5p, miR-93, and miR-223-3p, and downregulated ones such as miR-21-3p and miR-155-5p. Intriguingly, four miRNAs (miR-16-5p, miR-23a-3p, miR-30b, miR-191-5p) were identified to be significantly differentially expressed between patients suffering from dry and wet AMD (Ulańczyk et al., 2019) and could be served as potential biomarkers.

Likewise, Elbay et al. (2019) evaluated the serum miRNA levels of 120 subjects (70 wet AMD cases and 50 controls) and noticed an altered expression of 15 miRNAs (7 miRNAs were upregulated while 8 were downregulated) between AMD and control samples. Out of those 15 miRNAs, a significant upregulation of miR-486-5p and miR-626 and a downregulation of miR-885-5p in the AMD samples has been observed. Interestingly, several pathways, such as IGF1/AKT/mTOR, CD40, and mTOR, responsible for controlling angiogenesis, inflammatory responses, and photoreceptor degeneration have been regulated by miR-486-5p, indicating its crucial participation in wet AMD. At the same time, miR-626 has been identified as a suppressor of the SLC7A5 gene, a gene related to neuronal cell proliferation in the human brain, representing its possible role in the neurodegenerative process that occurs during AMD.

Elshelmani et al. (2020) performed a miRNA array in serum samples of 30 wet and dry AMD patients and 30 controls to identify the dysregulated miRNAs in either subtype of AMD or in both than that of the control group. 57 upregulated miRNAs were detected (46 in wet AMD, 4 in dry AMD, and 7 in both subtypes). Further validation with 14 out of the 57 dysregulated miRNAs revealed that three of them, miR-19a, miR-126, and miR-410, were substantially upregulated in both AMD subtypes as compared to control, indicating them as consistent biomarkers for AMD. Continuing with their previous study, Elshelmani et al. (2021) performed a bioinformatics analysis to determine the potential role of the three dysregulated miRNAs, obtaining more than 1,500 target genes involved in around 350 different pathways. In fact, while miR-126 was found to target ITGA6 and IGF-1 genes, which have a role in neurodegeneration, miR-410 and miR-19a were demonstrated to target several genes in the same pathways, such as NTRK1, IL1R1, PRKAR2B, PPP3CA, BIRC4, PIK3CA, MAP3K14, and PIK3R3 in apoptosis pathway; KRAS, MAPK1, PPP3CA, PIK3CA, NFATC2, SPHK2, PLA2G10, MAPK14, PIK3R3, and RAF1 in VEGF pathway; CD55, PLAU, CR2, and F3 in complement and coagulation cascade pathway; SOD1 in the neurodegenerative pathway; PEBF1 in nicotinate and nicotinamide metabolism; and ALDH1A2 in retinol metabolism.

It has been noticed that several signaling pathways are significantly affected by the dysregulated miRNAs in macular degeneration, including pathological angiogenesis and neurodegeneration (Figure 1C). Hence, further explicit functional elucidation of the associated miRNAs could be promising in AMD management.

## 5 Uveitis

Uveitis is the chronic inflammation of the uvea (the portion of the eye composed of the iris, retina, ciliary body, and choroid). It has been reported that in western countries, uveitis and its complications are responsible for about 10%–15% of preventable blindness (Bertrand et al., 2019). Uveitis can be classified into two types, infectious (IU) and non-infectious (NIU); moreover, NIU can be either idiopathic or caused by a systemic condition (Marino et al., 2019). Furthermore, uveitis can also be classified anatomically as anterior uveitides (affecting the anterior chamber), intermediate uveitides (affecting the vitreous), posterior uveitides (affecting the retina and/or choroid), and panuveitis (involving all the three aforementioned sites). A number of miRNAs have been observed to be differentially expressed in murine cells, participating in the development of this disease (Burkholder and Jabs, 2021).

Using autoimmune uveitis (EAU) mouse model, Zhang et al. (2020) demonstrated that miR-182-5p plays an important role in uveitis. CD4^+^T helper 17 cells (Th17), which secrete the cytokine interleukin (IL)-17, are relevant contributors to the development of uveitis and other autoimmune conditions. It has also been noticed that underexpressed miR-182-5p in EAU mouse-derived-Th17 cells negatively regulate its development. Moreover, miR-182-5p mimic therapy leads to the inhibition of Th17 cell development through targeting TATA-Box Binding Protein Associated Factor 15 (TAF15), mitigating the severity of EAU *in vivo.* Hence, the miR-182-5p/TAF15 axis could be a promising therapeutic target for uveitis (Zhang et al., 2020).

Later, Li et al. (2021) found that in the serum of EAU mice, miR-379-5p was significantly overexpressed, negatively regulating the expression of its potential target Semaphorin 3A (SEMA3A). SEMA3A is a secreted member of the semaphorin family, expressed in bones, kidneys, connective tissue, or neurons, and plays a crucial role in regulating the immune system. Moreover, SEMA3A has been related to lessening the effect of autoimmune diseases by diminishing the over-activity of B and T cells autoimmunity and regulating chemokines and cytokines. Nevertheless, the underlying molecular mechanism of miR-379-5p mediated regulation of SEMA3A in the pathogenesis of EAU is still elusive and needs further research (Li et al., 2021).

Other miRNAs related to anterior uveitis (AU) are miR-146a and miR-155. O’Rourke et al. (2019) observed that in human peripheral blood mononuclear cells (PBMC), the expression of these two miRNAs were significantly higher. Interestingly, the role of miR-146a was found to be regulatory since it targets and represses the expression of CD80, PRKCE, and VASN. CD80 is known to be a costimulatory molecule responsible for the T-cell proliferation, while the PRKCE is a constituent of TLR-4 signaling, substantially involved in AU pathogenesis and phosphorylating protein targets; VASN is a transmembrane protein linked to the inhibition of the transforming growth factor-beta (TGFβ), an immunosuppressive cytokine. The miR-155, on the other hand, has a pro-inflammatory role, negatively regulating SMAD2, TYRP1, and FBXO22. Downregulation of SMAD2 simultaneously decreases TGFβ at the gene transcription level, resulting in inflammation, while in AU, the increased expression of miR-155 diminishes the levels of FBXO22, leading to a loss of GogB function (an anti-inflammatory effector) and increased NF-κB activity, ultimately leading to inflammation. As well, TYRP1 is a melanocyte-specific gene product in melanin synthesis, and melanin has been identified as an inducer of AU. Furthermore, a feedback-loop relationship between miR-155a and miR-146a during the development of AU has also been suggested (O’Rourke et al., 2019).

The pathogenesis of uveitis has been linked to the dysregulated miRNAs associated with the immune system or inflammation (Figure 1D). Non-etheless, further research is necessary to establish the precise regulatory roles of each of the aforementioned miRNAs during the development of this disease so that they can be used for the management of the disease.

## 6 Conclusion

Eye diseases are some of the most significant and crucial disorders that affect an individual’s quality of life, either by causing complete blindness or vision impairment, but could be preventable if identified early. Eye diseases such as cataract, glaucoma, uveitis, and macular degeneration have shown numerous manifestations of altered miRNA expression. Research has provided substantial evidence about the role of miRNAs in the pathogenesis of these diseases by identifying multiple relevant miRNA targets whose deregulation leads to detrimental cell activities. Furthermore, the changes in the expression of circular miRNAs during the development and progression of eye diseases demonstrate their potential as non-invasive biomarkers for diagnosis and prognosis purposes.

In conclusion, even though the significant impact of miRNAs in eye disease development and their potential usage as biomarkers have been established, their more complex roles in pathogenesis are yet to be elucidated, which could be useful for novel miRNA-based drug development against chronic eye diseases such as cataract, glaucoma, uveitis, and macular degeneration.
